# Supplementary figures and images for: Direct Cryo-ET observation of platelet deformation induced by SARS-CoV-2 Spike protein
Source: bioRxiv. 2022 Nov 23:2022.11.22.517574. Preprint. [Version 1] doi: 10.1101/2022.11.22.517574 (PMC9709796; doi:10.1101/2022.11.22.517574)

**A**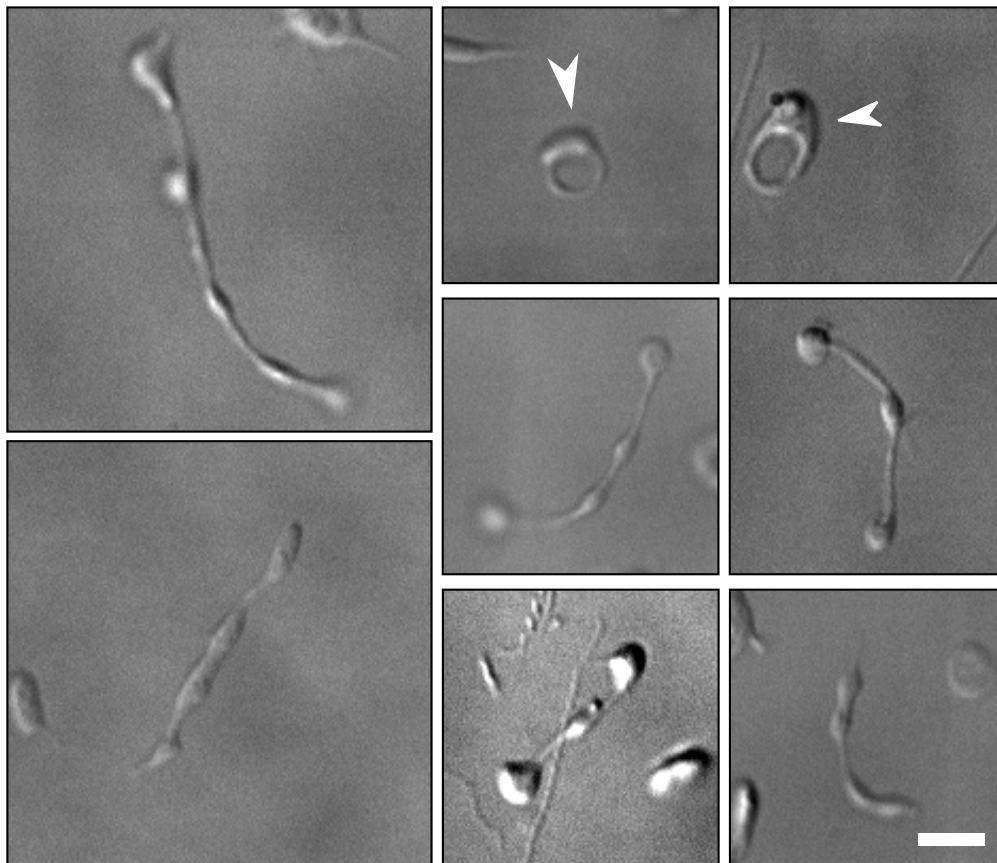**B**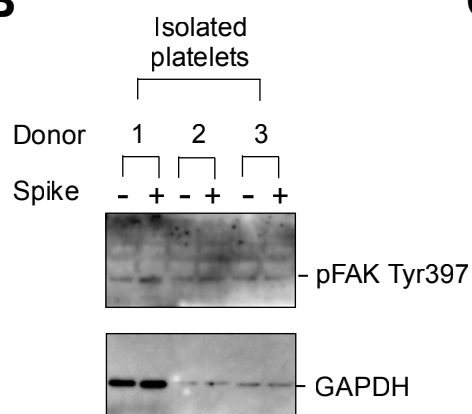**C**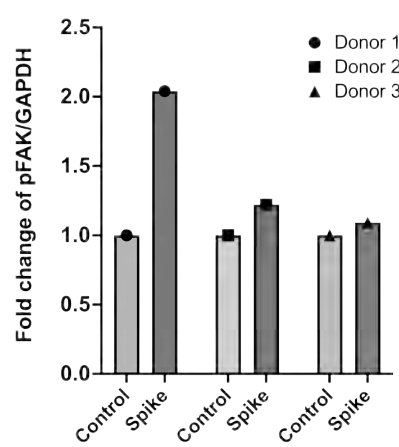**D**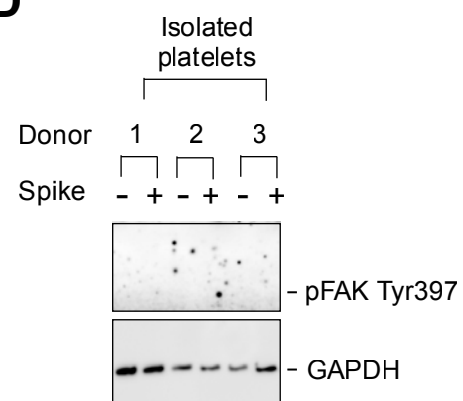

**A**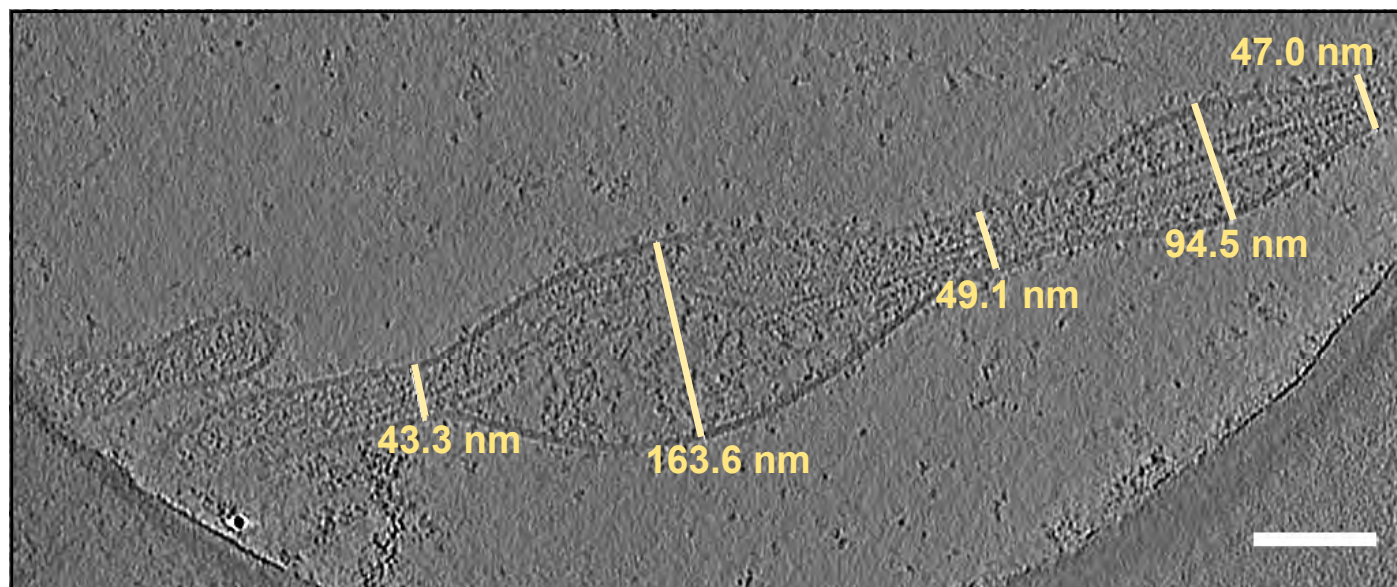**B**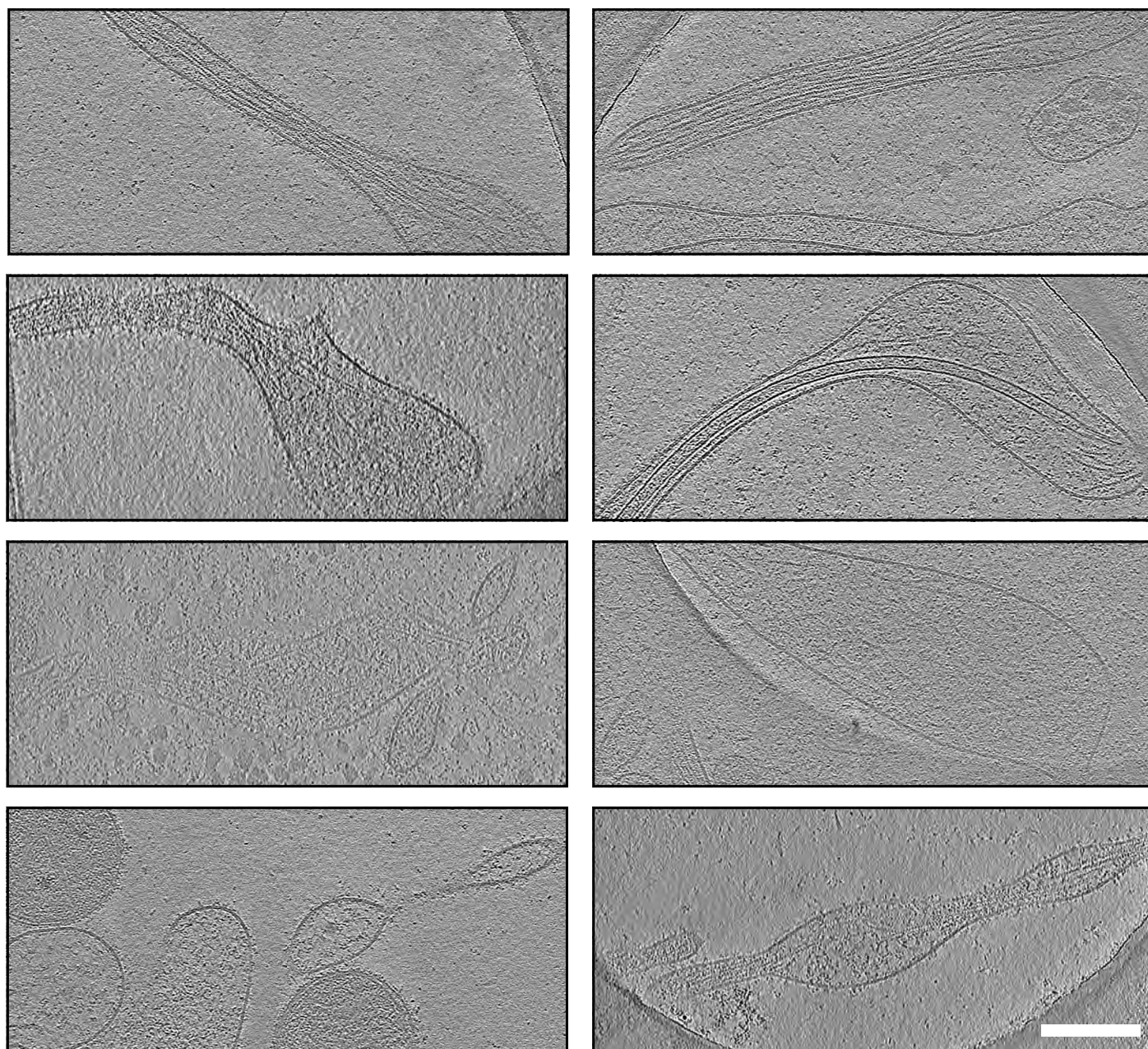

**A Open Form**

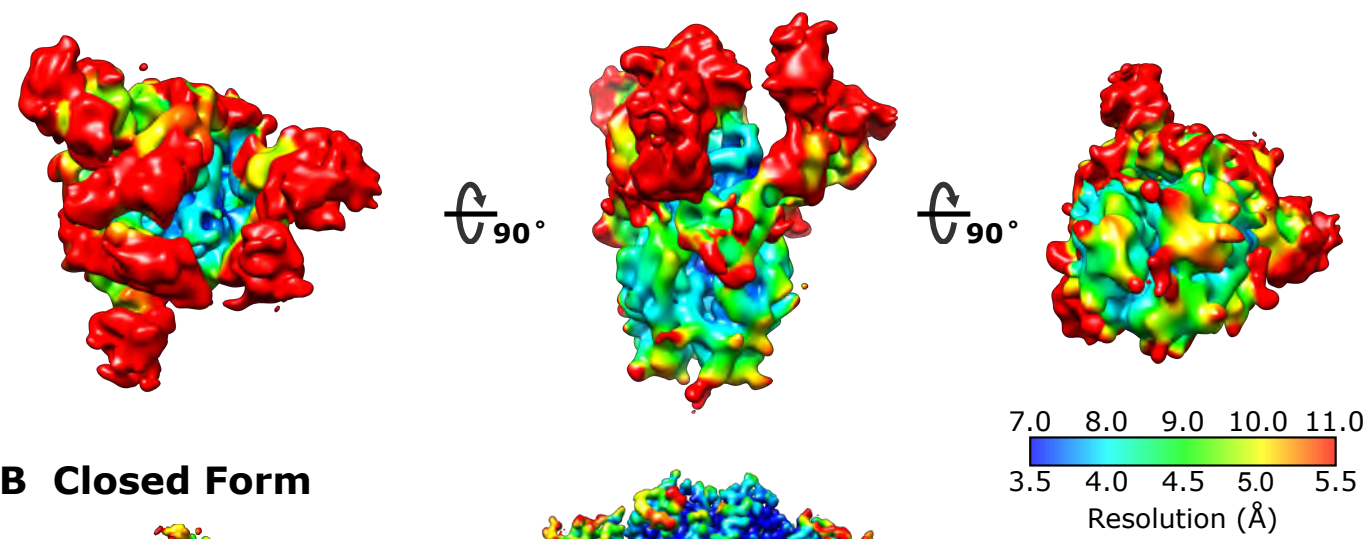

**B Closed Form**

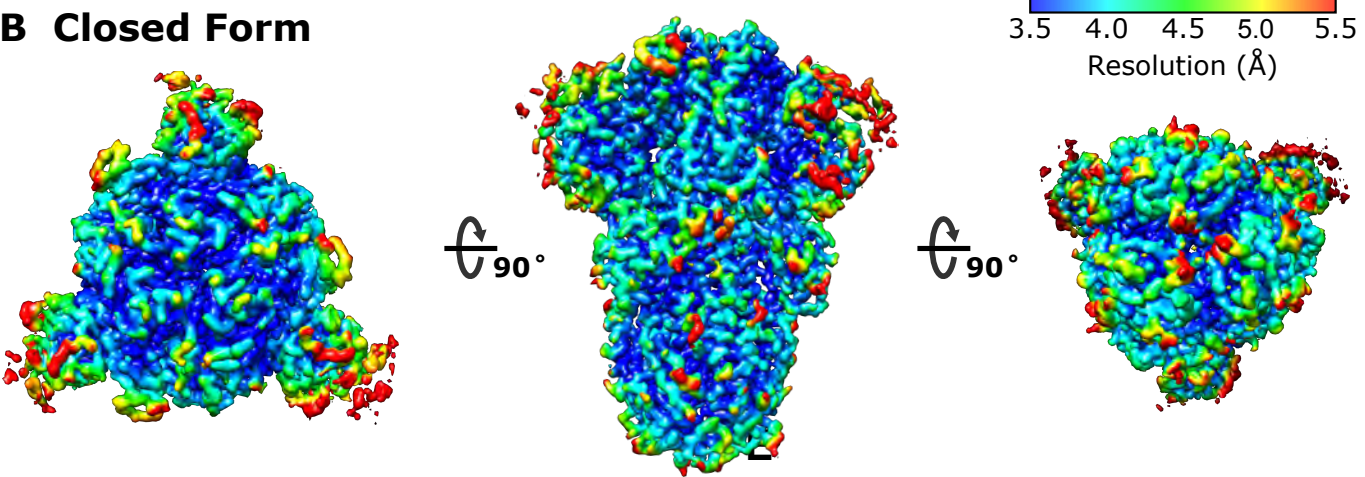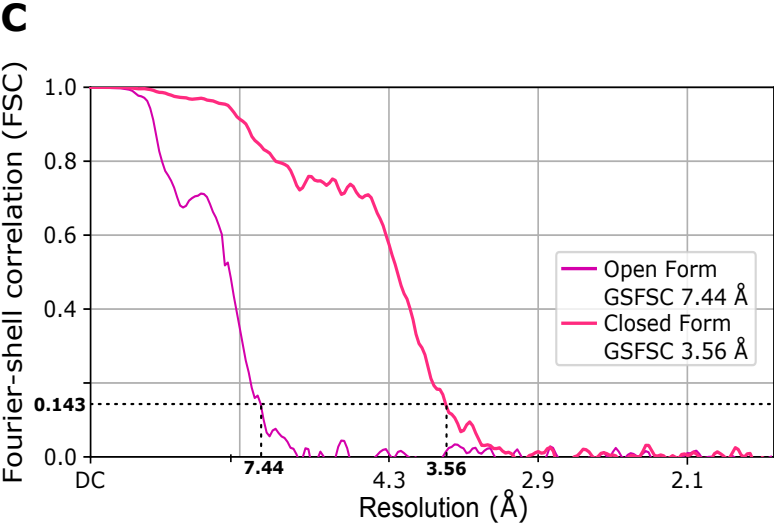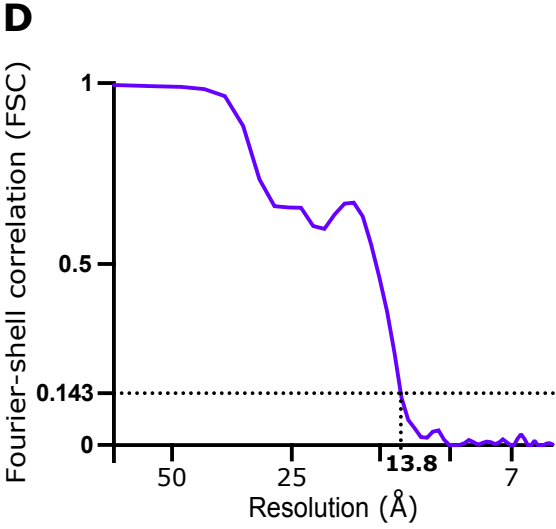

**A**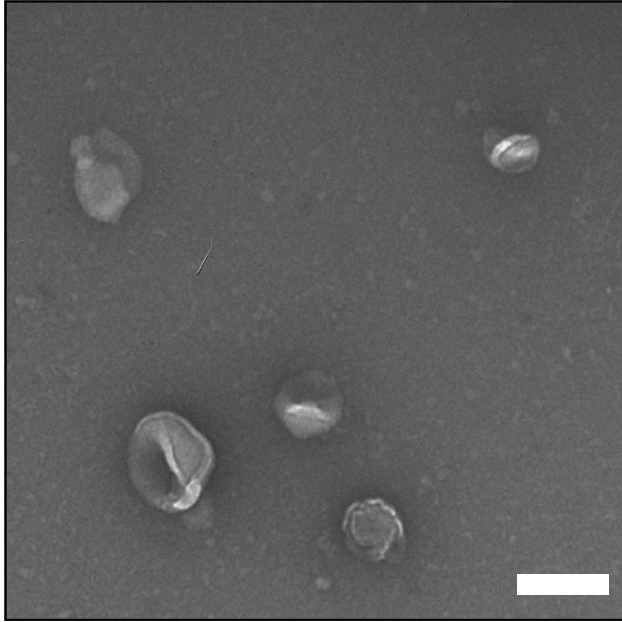**B**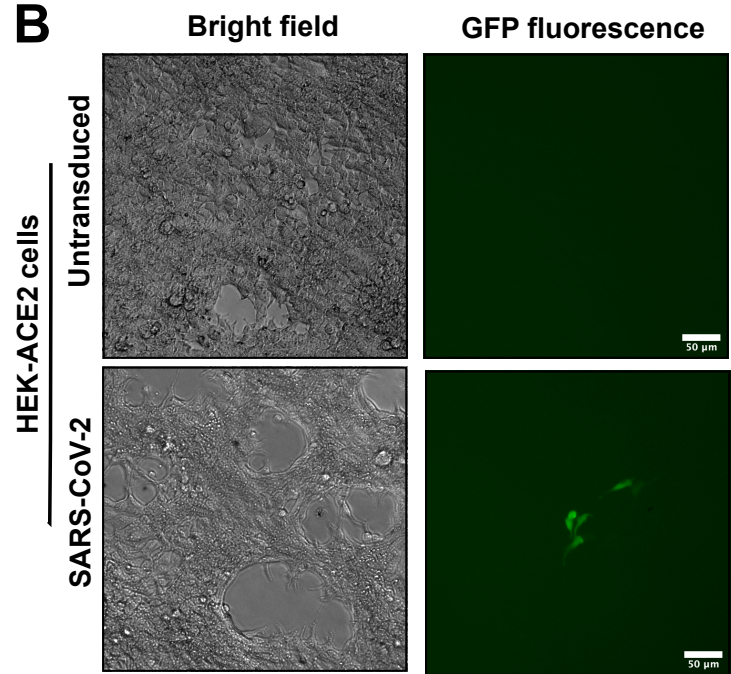**C**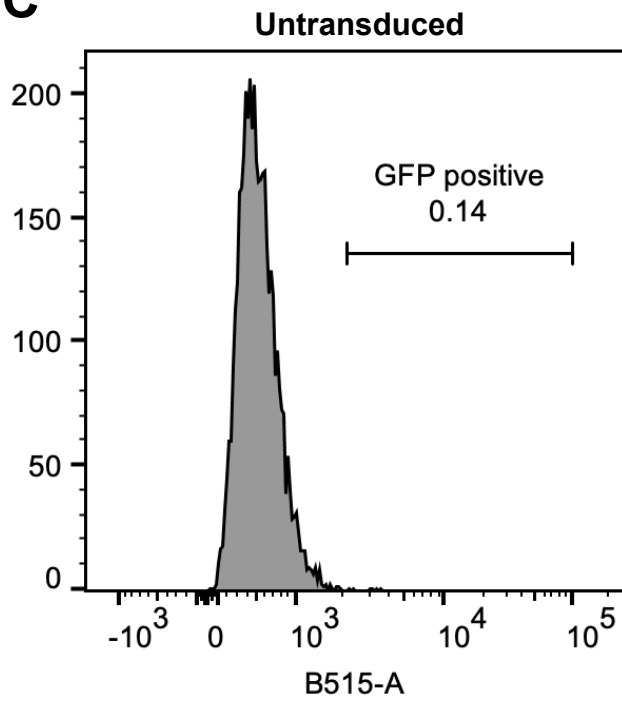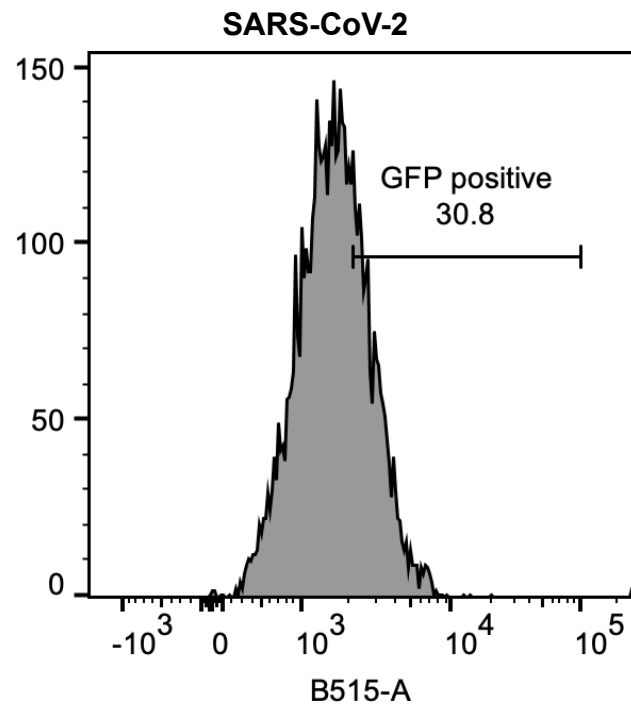

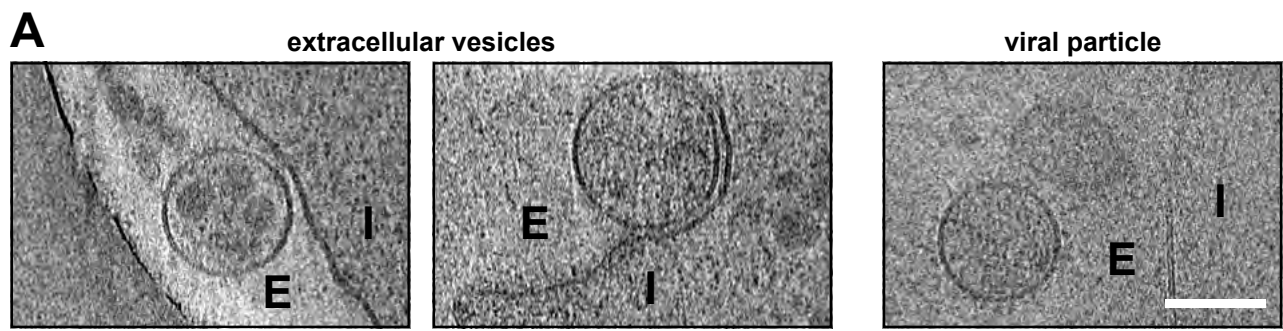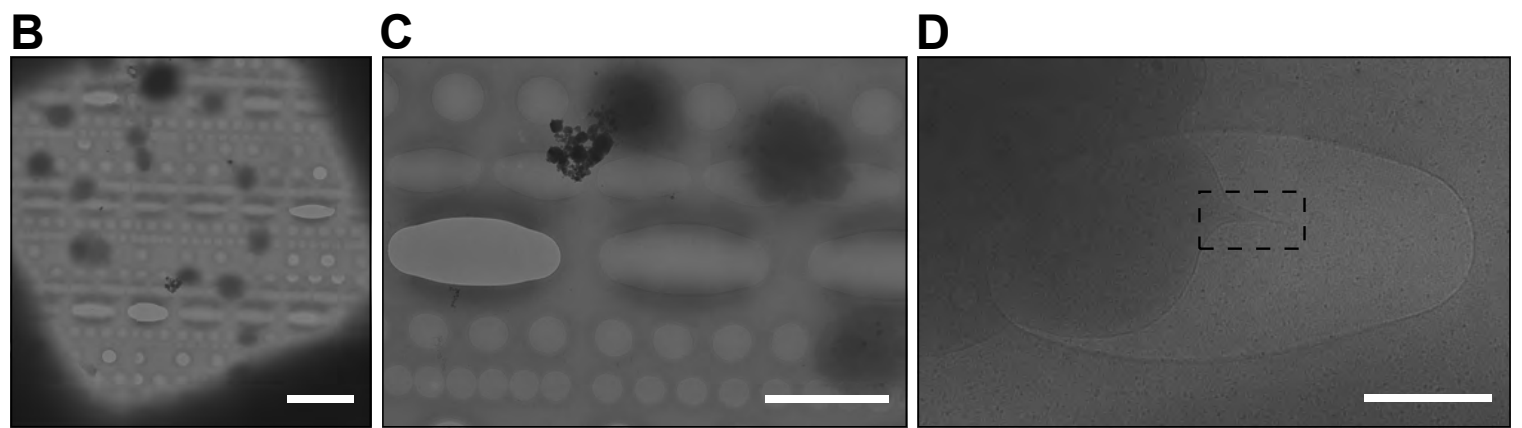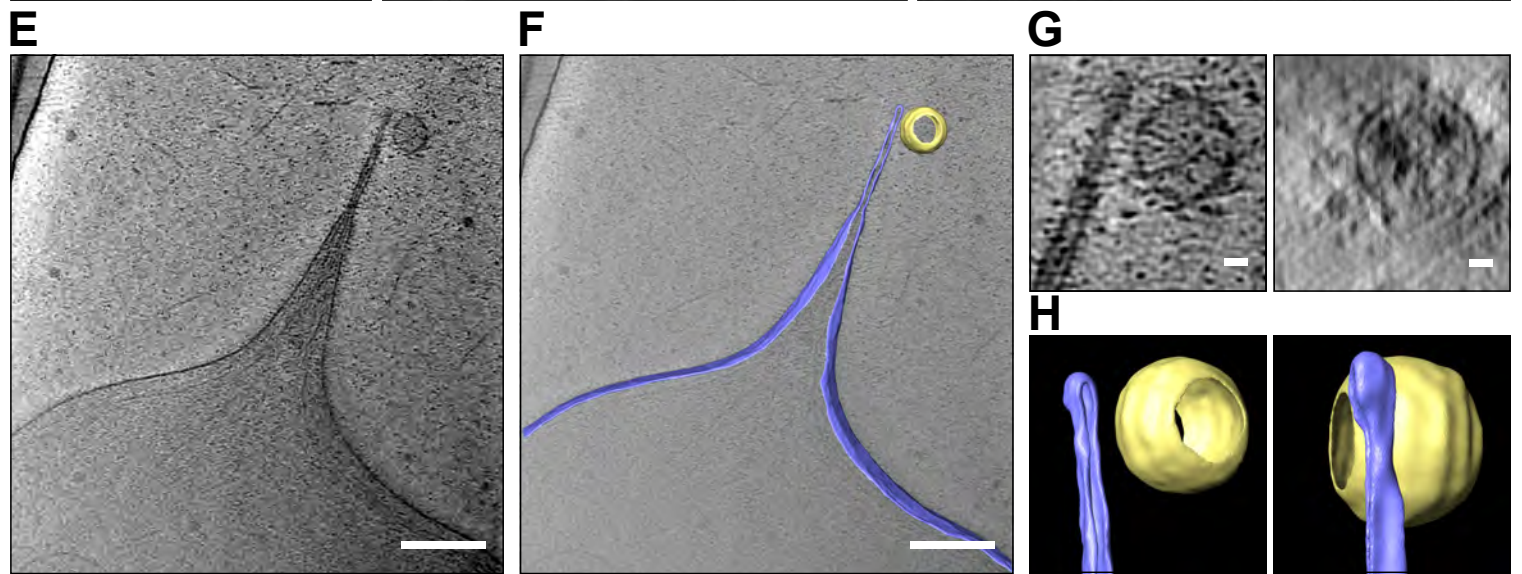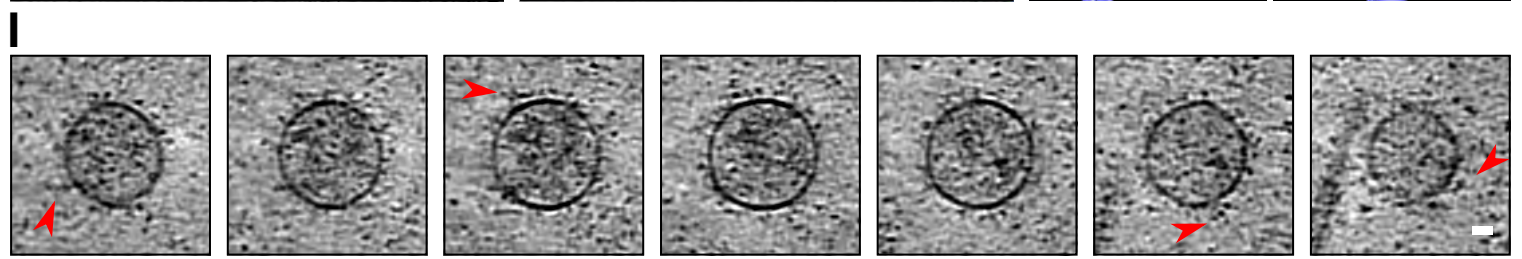

Supplement: Supplement 1 — Figure S1. Platelets incubated with SARS-CoV-2 S protein reveal proplatelet-like morphologies. (A) A subset of platelets showed a tubular appearance with multiple globular bodies along their elongated shape after the incubation with S protein. Some platelets had a ring-shaped appearance in the presence of the S protein. (B) The adherent platelets were collected in RIPA lysis buffer and the total concentration was quantified with Bradford assay. 30 μg of total protein was loaded on the gel and probed against pFAK or GAPDH. (C) Quantification of pFAK in adherent platelets normalized against GAPDH concentrations, showing the values of 2.04 (donor 1), 1.22 (donor 2) and 1.09 (donor 3). (D) The floating platelets were handled as specified in (B) and probed against pFAK, MLC or GAPDH. Scale bar: 5 μm. Figure S2. Cryo-electron tomographic visualization of platelets incubated exposed to SARS-CoV-2 S protein. (A) Filopodia width of the platelet incubated with S protein. (B) Central Slices of analyzed tomograms. The images show a slice through the deconvoluted tomogram used for further analysis. Scale Bars: (A) = 100 nm; (B) = 200 nm Figure S3. Cryo-EM structure of the SARS-CoV-2 S protein. (A) Cryo-EM map of S protein in the open conformation at a resolution of 7.44 Å. (B) Cryo-EM map of S protein in the closed conformation at a resolution of 3.56Å at FSC=0.143. (C) Gold standard FSC curves of the reconstructed S protein in the open and closed conformation. (D) (E) Gold standard FSC curve of the sub-tomogram averaged S protein reconstruction in the closed conformation with an estimated resolution of 13.8 Å at FSC=0.143. Figure S4. Characterization of SARS-CoV-2 S-pseudotyped lentiviral particles. (A) Negative-staining EM with 2% uranyl acetate of SARS-CoV-2 S-pseudotyped lentiviral particles. (B) Light microscopic images of HEK-hACE2 cells with or without treatment with pseudotyped lentivirus encoding ZsGreen. (C) Flowcytometry analysis of HEK-hACE2 cells transduced with pse [file media-1.pdf]
